# Functional oral nanoparticles for delivering silibinin and cryptotanshinone against breast cancer lung metastasis

**DOI:** 10.1186/s12951-020-00638-x

**Published:** 2020-05-30

**Authors:** Ying Liu, Xingmei Xie, Xuefeng Hou, Junyi Shen, Jiangpei Shi, Haizhen Chen, Yuanzhi He, Zhi Wang, Nianping Feng

**Affiliations:** School of Pharmacy, Shanghai University of Traditional Chinese Medicine, 1200 Cailun Road, Zhangjiang Hi-Tech Park, Pudong New District, Shanghai, 201203 People’s Republic of China

**Keywords:** Functional oral lipid-polymer hybrid nanoparticles, Metastasis, Mucus penetration, Silibinin, Cryptotanshinone, Tumor microenvironment

## Abstract

**Background:**

Breast cancer lung metastasis occurs in more than 60% of all patients with breast cancer, and most of those afflicted by it eventually die of recurrence. The tumor microenvironment plays vital roles in metastasis. Modulating the tumor microenvironment via multiple pathways could efficiently prevent or inhibit lung metastasis. Silibinin and cryptotanshinone are natural plant products that demonstrate anti-metastasis effects and modulate the tumor microenvironment via different pathways. However, they have poor aqueous solubility, membrane permeability, and oral bioavailability. Oral drug administration may help improve the quality of life and compliance of patients with breast cancer, primarily under long-term and/or follow-up therapy. Herein, we developed poly-N-(2-hydroxypropyl) methacrylamide (pHPMA)-coated wheat germ agglutinin-modified lipid-polymer hybrid nanoparticles, co-loaded with silibinin and cryptotanshinone (S/C-pW-LPNs). We assessed their oral bioavailability, and evaluated their anti-metastasis efficacy in a 4T1 breast cancer tumor-bearing nude mouse model.

**Results:**

An in vitro mucus diffusion study revealed that pHPMA enhanced W-LPN mucus penetration. After oral administration, pHPMA enhanced nanoparticle distribution in rat jejunum and substantially augmented oral bioavailability. S/C-W-LPNs markedly increased 4T1 cell toxicity and inhibited cell invasion and migration. Compared to LPNs loaded with either silibinin or cryptotanshinone alone, S/C-pW-LPNs dramatically slowed tumor progression in 4T1 tumor-bearing nude mice. S/C-pW-LPNs presented with the most robust anti-metastasis activity on smooth lung surfaces and mitigated lung metastasis foci. They also downregulated tumor microenvironment biomarkers such as CD31, TGF-β1, and MMP-9 that promote metastasis.

**Conclusions:**

Silibinin- and cryptotanshinone-co-loaded pW-LPNs efficiently penetrate intestinal barriers, thereby enhancing the oral bioavailability of the drug loads. These nanoparticles exhibit favorable anti-metastasis effects in breast cancer-bearing nude mice. Hence, S/C-pW-LPNs are promising oral drug nanocarriers that inhibit breast cancer lung metastasis.

## Background

Breast cancer metastasis is responsible for high mortality among women worldwide [1, 2]. The lungs are primary targets in breast cancer metastasis. Moreover, there is a high rate of recurrence in lung metastasis [3]. The tumor microenvironment consists mainly of blood vessels, endothelial fibroblasts, immune cells, macrophages, signaling molecules, and extracellular matrix (ECM). The associations between tumor cells and the other components in the tumor microenvironment influence metastasis. Therefore, the components of the tumor microenvironment are, in fact, allies of cancer progression [4, 5]. Drug delivery systems associated with the tumor microenvironment may enhance anti-metastasis efficiency [6]. Nanocarriers can modulate the tumor microenvironment and inhibit metastasis. Anti-metastasis via conventional chemotherapy often fails as the drugs might induce multiple systemic side effects. Furthermore, the therapeutic agents may be inefficient as they cannot contend with the complex pathophysiology and multiple processes involved in metastasis. Alternative treatments derived from natural plant products could provide enhanced anti-metastatic efficacy while inducing fewer side effects.

Silibinin (SLB) is a flavonolignan derived from the fruit of *Silybum marianum* (L.) Gaertner. In the tumor microenvironment, SLB [7, 8] inhibits tumor angiogenesis [9] and negatively regulates the epithelial-mesenchymal transition (EMT) [7]. It reduces the interaction between ECM and tumor cells by repressing matrix metalloproteinase (MMP) and vascular endothelial growth factor (VEGF) [10]. Silybin phospholipid complex (Siliphos^®^), administered orally to early-stage breast cancer patients, concentrates in breast cancer tissues and appears at low levels in normal tissues [11]. Thus, orally administered SLB can regulate breast cancer tumor microenvironment in vivo. Cryptotanshinone (CT) is a quinoid diterpene derived from *Salvia miltiorrhiza* Bunge. It induces tumor apoptosis, inhibits cancer cell proliferation, and modulates EMT [12–16]. As breast cancer metastasis is complex, we hypothesized that the induction of anti-metastasis via different biochemical pathways could simultaneously augment an anti-metastasis effect. A single formulation, co-loaded with SLB and CT, could accomplish this task. However, these substances may have low solubility, limited intestinal absorption, short elimination time, and poor in vivo bioavailability.

Recently, novel oral anti-cancer and anti-metastasis drug delivery systems have been developed [17–19]. It was reported that several nanocarriers such as maleimidyl-poly(ethylene glycol)-*co*-poly(ε-caprolactone) and poloxamer P188 nanoparticles improve the bioavailability and anti-metastasis efficacy of oral drugs in vivo [20, 21]. Oral nanocarriers ameliorate the quality of life and compliance of the patients especially under long-term treatment and follow-up therapy [19, 22]. However, the intestinal mucus layer and enterocytes are nanocarrier diffusion and transport barriers. In our previous study, we reported the development of wheat germ agglutinin-modified lipid-polymer hybrid nanoparticles (WGA-LPNs) coated with polyethylene glycol (PEG). The WGA modification promotes in vitro cancer cell uptake via receptor-mediated endocytosis. It targets *N*-acetyl-*d*-glucosamine and sialic acid on the surfaces of enterocytes and microfold cells. PEGylation augments nanoparticle penetration through the mucus layer and prevents the mucin from binding to WGA [23]. The biocompatible hydrophilic polymer poly-N-(2-hydroxypropyl) methacrylamide (pHPMA) can detach from the surfaces of the nanoparticles so that they are available for absorption and endocytosis [24, 25]. Thus, nanoparticle coatings facilitate mucus penetration without compromising cellular uptake. Hence, we propose that pHPMA could serve as a protective, detachable W-LPN coating.

Herein, SLB and CT were co-loaded into pHPMA-coated W-LPNs (S/C-pW-LPNs) to improve the oral bioavailability and anti-metastasis effects of these drugs. We investigated the mucosal diffusion behavior and cellular endocytosis mode of action and the influence of mucin on endocytosis. We also evaluated the anti-metastasis efficacy of these nanoparticles on 4T1 breast cancer cells and a 4T1 tumor-bearing nude mouse model. We attempted to elucidate the in vivo tumor microenvironment alteration mechanism of S/C-pW-LPNs in terms of angiogenesis, epithelial-to-mesenchymal transition, and ECM remodeling.

## Results

### Preparation and characterization of pW-LPNs

**Fig. 1:**
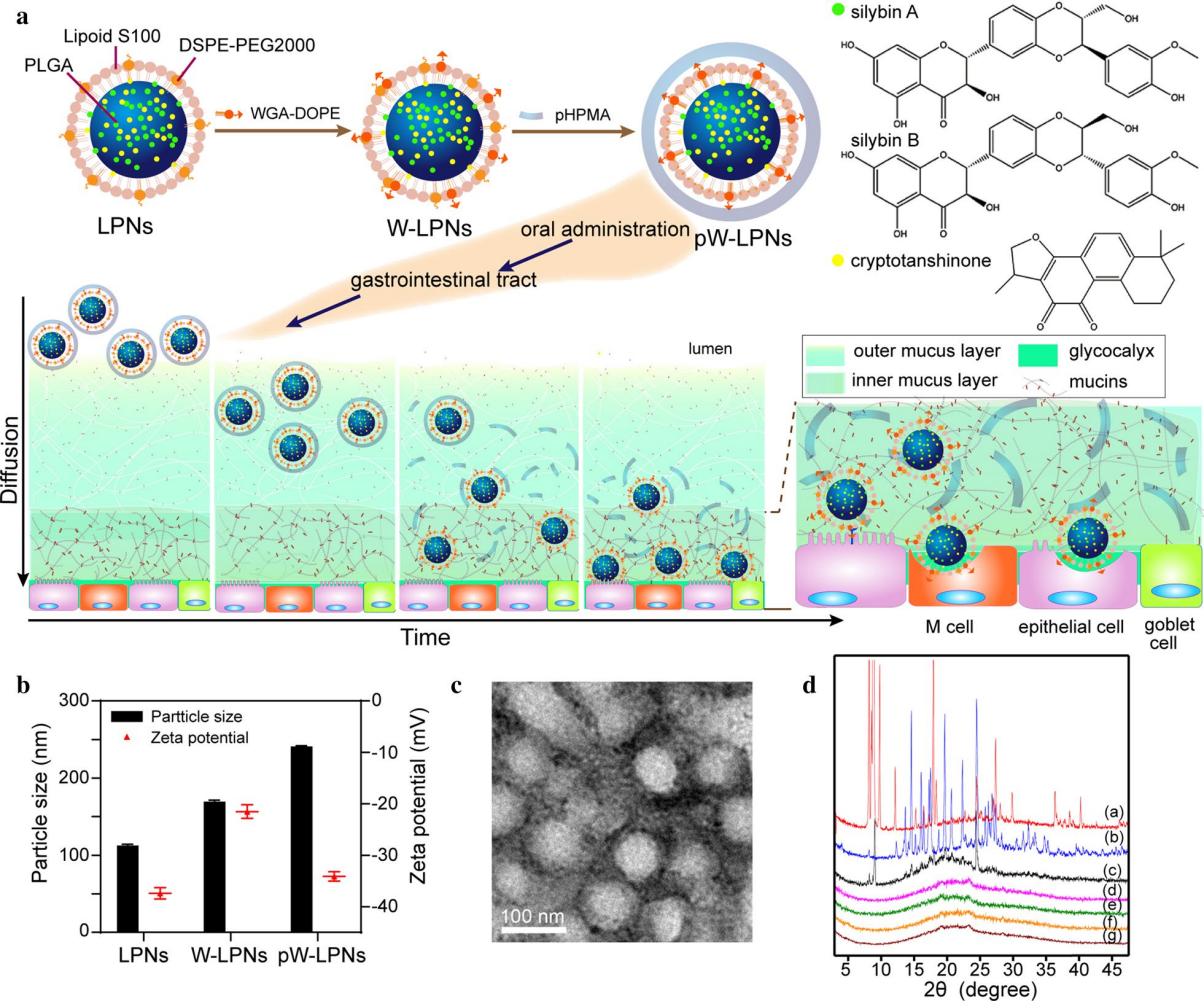
**a** Schematic diagram of S/C-pW-LPN construction and its putative fate during mucus penetration and endocytosis. Silybin A and silybin B are the main components of silibinin. **b** Particle sizes and zeta potentials of LPNs, W-LPNs, and pW-LPNs (mean ± SD, n = 3). **c** Transmission electron microscopy image of pW-LPNs (scale bar = 100 nm). **d** X-ray powder diffraction spectra of (a) pure cryototanshinone; (b) pure silibinin; (c) physical mixture; (d) blank LPNs; (e) S/C-LPNs; (f) S/C-W-LPNs; and (g) S/C-pW-LPNs

The pW-LPNs were prepared as shown in Fig. 1a. The LPNs were developed using a modified nanoprecipitation technique that encapsulated SLB and CT. The W-LPNs were formed via WGA-DOPE insertion and coated with hydrophilic, biocompatible pHPMA polymer.

The pW-LPNs had greater hydrodynamic diameters than the LPNs (Fig. 1b). Transmission electron microscopy (TEM) revealed that the pW-LPNs were spherical (Fig. 1c). X-ray powder diffraction (XRD) analysis determined SLB and CT encapsulation in the nanoparticles by detecting crystalline changes. Figure 1d indicates that pure SLB and CT were highly crystalline and had numerous diffraction peaks. The pure CT had distinctive peaks at 9.0°, 9.8°, 12.1°, 17.2°, 24.9°, 27.3°, and 29.8°, whereas pure SLB had distinctive peaks at 14.6°, 17.5°, 19.6°, 22.3°, and 24.5°. For the mixture, certain peaks were weak because physical mixing may have altered the crystalline properties of SLB and CT. The absence of SLB and CT peaks in the pW-LPNs suggested that the SLB and CT dispersed within the nanoparticles might be in amorphous or molecular form and/or in a disordered crystalline phase.

### pHPMA enhanced W-LPN diffusion through mucus

**Fig. 2:**
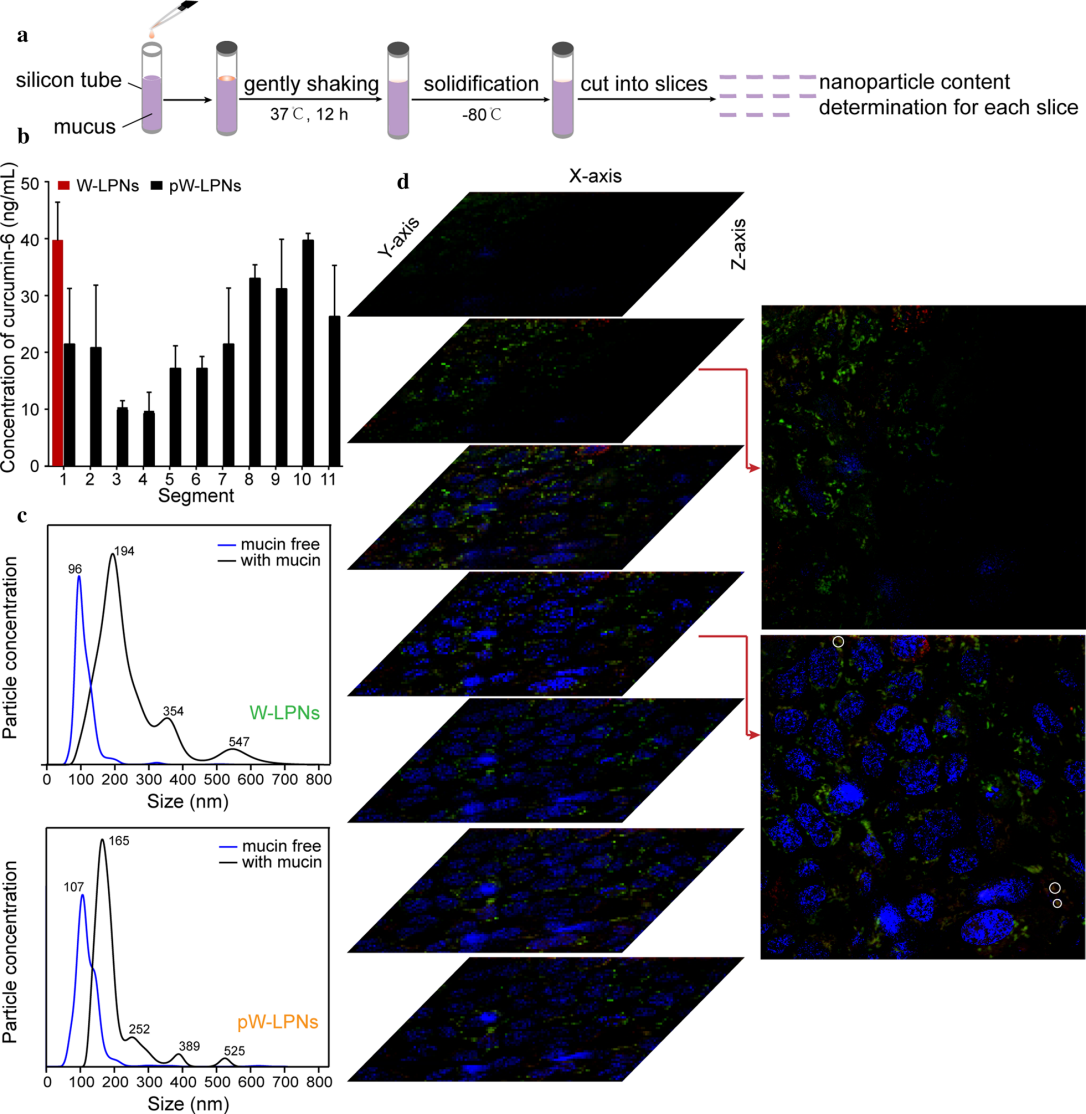
**a** Schematic illustration for silicon tube rotation test. **b** W-LPNs and pW-LPNs diffusion through porcine mucus. **c** Concentration-to-size nanoparticle distribution before (blue curves) and after (black curve) 30 min incubation. **d** Confocal images showing pW-LPN diffusion and penetration through the mucus secreted by HT29-MTX-E12 cells. Double fluorescent-labeled nanoparticles with TRITC-labeled pHPMA (red signal) and FITC-labeled nanoparticles (green signal) were applied to the cell surface for 1 h. Sequential stacked xy-plane (rotation angles: x = 80°, y = 18°, z = 270°) in z-series at 10-μm intervals parallel to cell growth on glass bottom

pW-LPN diffusion was performed using a silicon tube rotation test to evaluate the ability of pHPMA to enhance nanoparticle mucus penetration (Fig. 2a). Figure 2b shows that pW-LPNs were distributed throughout the segments, whereas W-LPNs were distributed only in the first segment.

Nanosight tracking assay was conducted using quantum dot (QD)-labeled nanoparticles to establish whether the enhancement of mucus penetration was associated with the interactions between nanoparticles and mucin and the underlying mechanism. This procedure was performed to avoid intrinsic fluorescence interference. Mucin-free pW-LPNs and W-LPNs exhibited normal, narrow size distributions mainly < 300 nm (Fig. 2c). After incubation with mucin for 30 min, the peak particle size of W-LPNs increased from 96 nm to 194 nm and the size distribution range expanded. In contrast, the pW-LPNs had relatively narrower particle size distributions. Moreover, the concentration and intensity of W-LPNs particles > 300 nm were higher than those of pW-LPNs, possibly because of agglomeration and adhesion of W-LPNs to mucins and other components. The results suggest that these nanoparticles had different mucin binding capacities and the presence of pHPMA reduced mucin binding and facilitated nanoparticle diffusion through the mucus.

Confocal laser scanning microscopy (CLSM) was performed to investigate pW-LPN distribution and diffusion behavior through the mucus layer formed by HT29-MTX-E12 (E12) cells double-labeled with tetramethylrhodamine (TRITC)-labeled pHPMA and fluorescein isothiocyanate (FITC)-labeled W-LPNs. Micrographs on the xy-plane were arranged in z-series tiers at a 10-μm step depth. Figure 2d shows the stacked rotated images. The second image represents the location at ~ tens of micrometers from the nucleus. The clear and strong red and bright green fluorescent signals indicated that pHPMA detached from the nanoparticles. The fourth image represents the inner side of the cell and shows bright green signals indicating that the pHPMA-free nanoparticles were widely distributed throughout the cytoplasm. However, W-LPNs associated with pHPMA sporadically appeared (white circle). Hence, pHPMA could detach from the W-LPNs during mucus layer diffusion. After penetration through the mucus layer, the nanoparticles were exposed to the cell membranes, which led to absorption. In the subsequent cellular uptake study, endocytosis of W-LPNs was evaluated.

### Cellular uptake

**Fig. 3:**
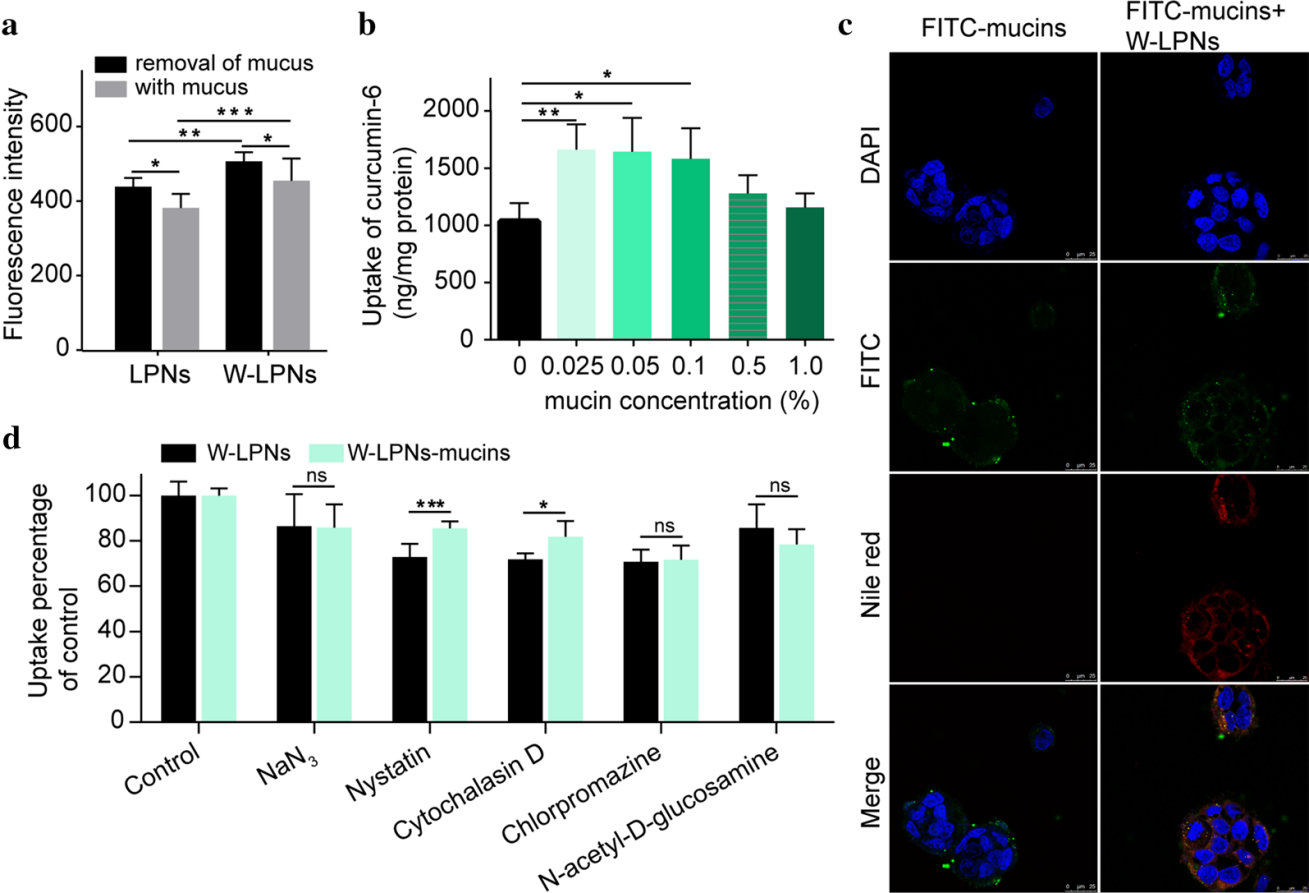
**a** Cellular uptake of LPNs and W-LPNs in a Caco-2/HT29-MTX-E12 co-culture with or without mucus treatment with *N*-acetyl-*l*-cysteine. **b** Quantities of coumarin-6 absorbed per milligram cellular protein in Caco-2 cells treated with WGA-LPNs after co-incubation with mucin at various concentrations. **c** Confocal microscopy images of Caco-2 cells treated with FITC-labeled mucins and WGA-LPNs after incubation with FITC-labeled mucins. Rows 1–4 show the DAPI channel, fluorescein isothiocyanate (FITC) channel, tetramethylrhodamine isothiocyanate (TRITC) channel, and merged images, respectively. **d**  % uptake of WGA-LPNs or WGA-LPN-mucins by Caco-2 cells *vs* control in the presence of different inhibitors (mean ± SD, n = 5). *P < 0.05, **P < 0.01, ***P < 0.001

Cellular uptake rates are shown in Fig. 3a. The W-LPN fluorescence intensities in Caco-2/E12 co-culture cells (3:1) were higher than those in the LPNs. After removal of mucus with *N*-acetyl-*l*-cysteine, the W-LPN fluorescence intensities were greater than the LPNs. This finding aligned with that of a previous report [26] and indicated that WGA modification might enhance cellular LPN uptake.

The influence of mucin on cellular W-LPN uptake was investigated by loading mucin labeled with FITC and Nile Red into W-LPNs and visualizing cellular uptake using CLSM (Fig. 3c). The images reveal intracellular free FITC-labeled mucin distribution. For the W-LPNs with mucin, green and red signals were distributed around the nucleus. Thus, the cells may have internalized W-LPN-mucin agglomerates. For the cells subjected to W-LPNs preincubated with mucin, the Nile Red fluorescence intensity was more potent than the mucin-free W-LPNs. It should be noted that Nile Red may stain the lipid components, and quantitative analysis was conducted to detect the influence of mucin on cellular uptake. The quantitative analysis of cellular uptake showed that at a mucin concentration range of 0.025–0.1%, mucin preincubation enhanced W-LPNs uptake, whereas cellular W-LPN uptake efficiency was not concentration-dependent (Fig. 3b). Further, W-LPNs preincubated with 0.5–1% mucin presented with cellular uptake comparable to that of mucin-free W-LPNs.

To elucidate the endocytosis mechanism, we compared the cellular uptake of mucin-free W-LPNs and those preincubated with mucin in the presence of the endocytic pathway inhibitors sodium azide (NaN_3_), nystatin, cytochalasin D, and chlorpromazine. As endocytic pathways are generally energy-dependent, cellular uptake was evaluated in the presence of the energy-depleting agent NaN_3_. Figure 3d shows that the relative cellular W-LPN uptake with or without mucin decreased by 85.8 ± 10.1% and 86.4 ± 1.2%, respectively. Nystatin inhibits caveolar endocytosis [27]. Here, it impeded W-LPN uptake with and without mucin preincubation. Cellular uptake of W-LPNs preincubated with mucin was significantly higher than that of W-LPNs without mucin pretreatment (*P* < 0.001). Cytochalasin D inhibits micropinocytosis-mediated endocytosis [27]. It considerably reduced W-LPNs preincubated with or without mucin and lowered the inhibitory action of W-LPNs preincubated with mucin compared to that of W-LPNs preincubated without mucin. Cellular uptake of W-LPN preincubated with and without mucin was blocked by chlorpromazine treatment. *N*-acetyl-*d*-glucosamine (NAG) was used as a competitive inhibitor of WGA binding to Caco-2 cells. NAG reduced cellular W-LPN uptake in the presence and absence of mucin. Further, WGA receptor-mediated endocytosis occurred.

### Cell viability assay and in vitro anti-metastasis study

**Fig. 4:**
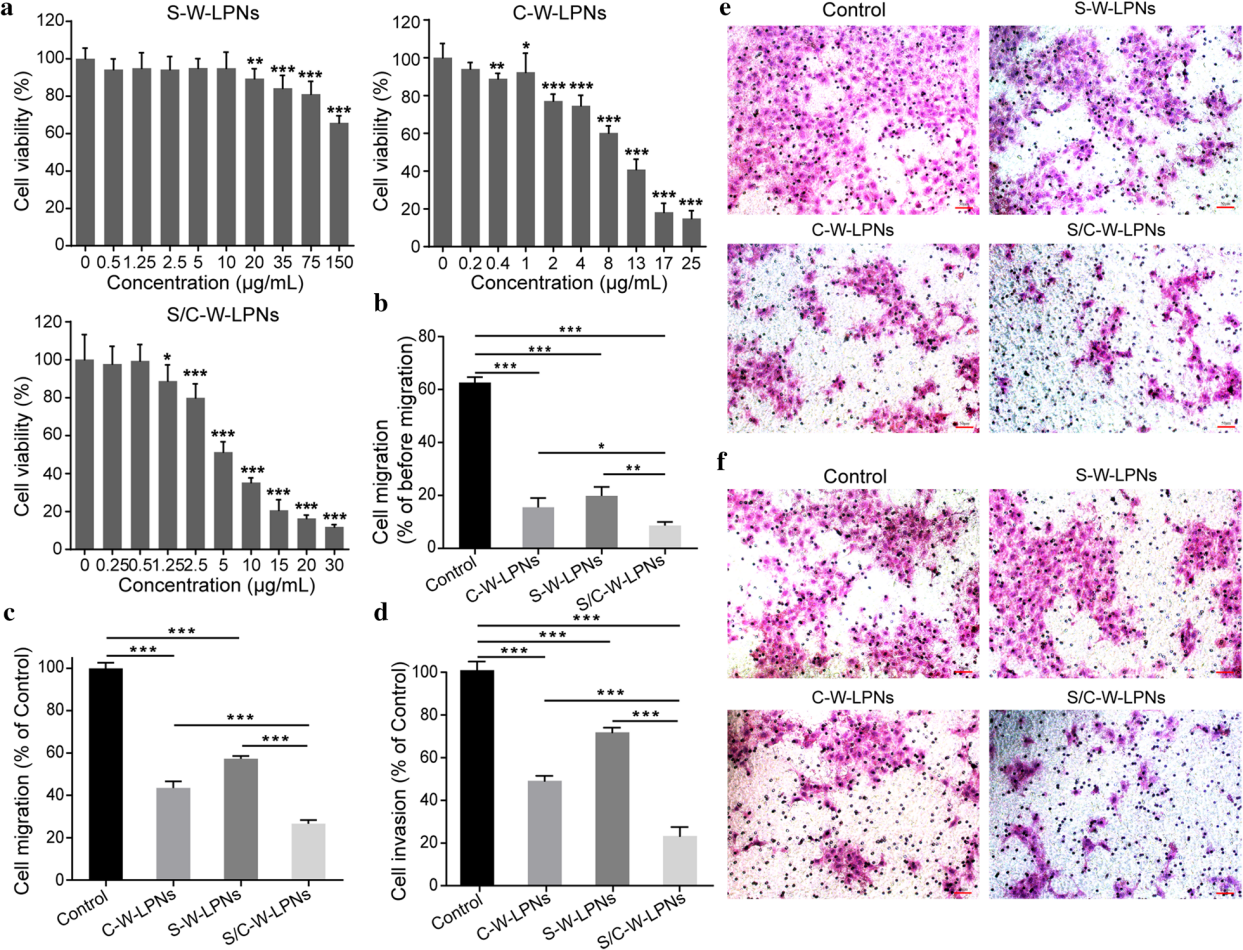
**a** Cytotoxicity effects on 4T1 cells treated with S-W-LPNs, C-W-LPNs and S/C-W-LPNs. **b**–**f** 4T1 cell metastasis inhibition assay. **b**–**d** Semiquantitative analysis; **b** Cell migration in wound healing assay. **c** Transwell assay. **d** Matrigel invasion assay (mean ± SD, n = 3). **e** Hematoxylin-eosin-staining images of Transwell migration assay. **f** Hematoxylin-eosin-staining images of Matrigel invasion assay. *P < 0.05, **P < 0.01, ***P < 0.001

The cytotoxicity of S/C-W-LPN, S-W-LPN, and C-W-LPN in 4T1 cells was determined using Cell Counting Kit 8 (CCK-8, Sigma-Aldrich Co., MO, USA) assay. As shown in Fig. 4a, the cells treated with 75 μg/mL S-W-LPNs had > 80% viability. The IC_50_ for C-W-LPNs and S/C-W-LPNs were 8.30 μg/mL and 5.94 μg/mL, respectively.

The anti-metastatic effects of SLB/CT-coloaded W-LPNs were evaluated by determining the motility and interactions of highly metastatic 4T1 cells with C-W-LPNs, S-W-LPNs, and S/C-W-LPNs. After treatment with C-W-LPNs, S-W-LPNs, and S/C-W-LPNs, the relative cellular migration rates were 15.5 ± 3.58%, 19.9 ± 3.35%, and 8.6 ± 1.38%, respectively (Fig. 4b).

A Transwell migration assay was conducted to confirm the effects of S/C-W-LPNs on longitudinal 4T1 cell motility. Figure 4e reveals that relative to the control, 4T1 cells treated with C-W-LPNs, S-W-LPNs, and S/C-W-LPNs presented with considerably less migration activity. Only 26.7 ± 1.59% of the 4T1 cells exposed to S/C-W-LPNs passed through the membrane (Fig. 4c). This migration rate was dramatically lower than that measured for the 4T1 cells subjected to C-W-LPNs and S-W-LPNs. We also performed an invasion assay on a Matrigel-coated Transwell to determine the metastasis-promoting capacity of ECM barrier migration. After treatment with C-W-LPNs, S-W-LPNs, and S/C-W-LPNs, 48.5 ± 2.4%, 71.1 ± 2.2%, and 22.9 ± 4.2% of the cells passed through the Matrigel (Fig. 4d, f).

### pW-LPN distribution in the rat intestine

**Fig. 5:**
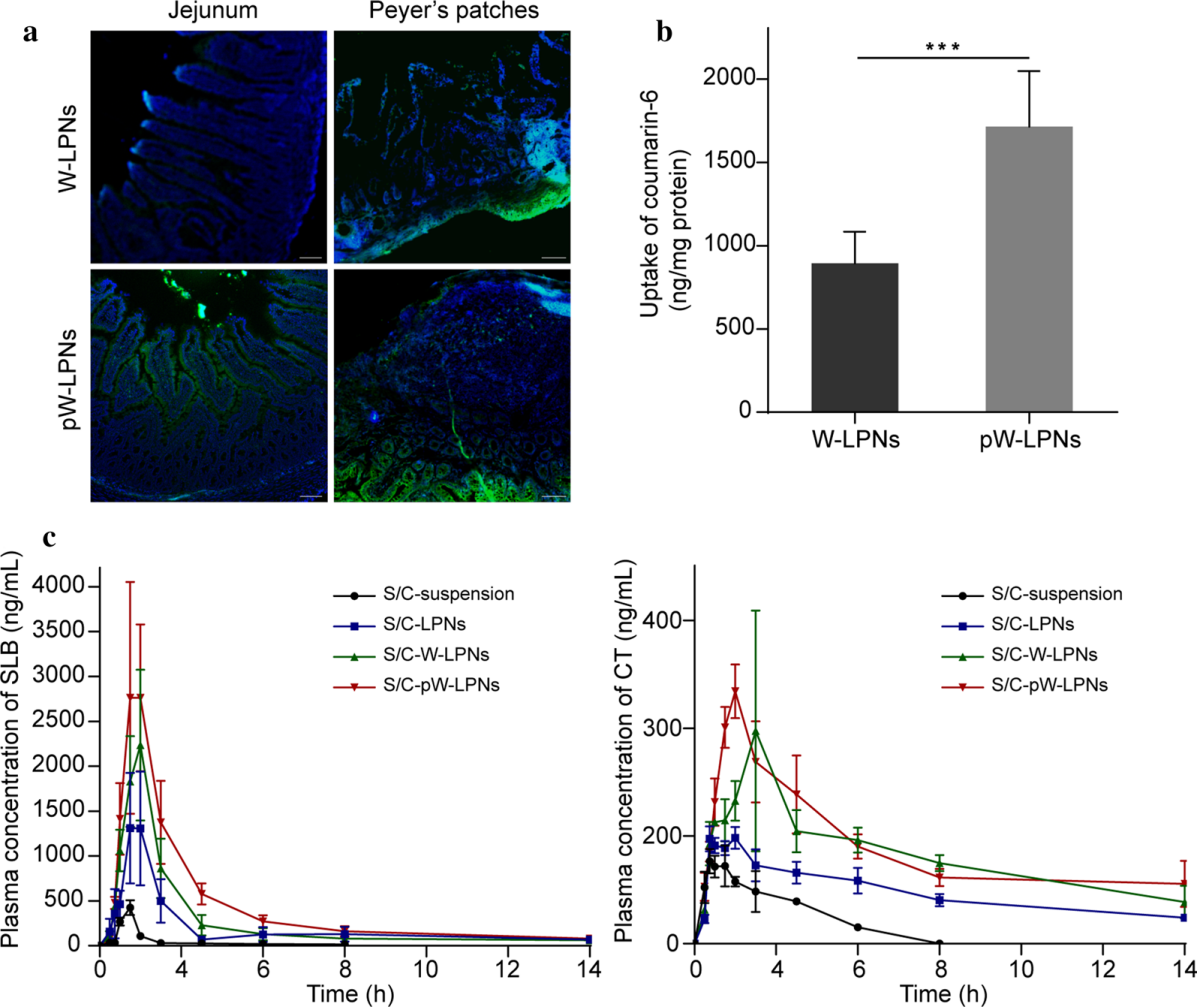
**a** Typical distributions of W-LPNs and pW-LPNs in rat jejunum and typical particle absorption in Peyer’s patches after oral gavage. (scale bar = 100 μm). **b** Quantitative analysis of particles in Peyer’s patch tissue (mean ± SD, n = 3). **c** S/C-suspension, S/C-LPNs, S/C-W-LPNs, and S/C-pW-LPNs concentration–time curves for SLB (left) and CT (right) in rats following *ig* administration at 80 mg/kg (mean ± SD, n = 5). ***P < 0.001

pW-LPN distribution assay in the rat intestine was performed to explore whether orally administered pHPMA-nanoparticle associations could influence nanoparticle distribution in the jejunum in rats. Figure 5a show fluorescence microscopy images of jejunum cross-sections from rats fed with W-LPNs and pW-LPNs. One hour after the administration, all nanoparticles were observed in the jejunum. Compared with the W-LPNs, pW-LPNs had stronger green fluorescence signals and were, therefore, more widely distributed over the tissue surface. Moreover, the pW-LPNs were evenly distributed between the villi. Finally, the pW-LPNs more deeply penetrated the epithelia and intestinal cells than the W-LPNs.

The nanoparticle distributions in rat intestinal Peyer’s patches was performed to evaluate the influence of the pHPMA-nanoparticle associations on alternative absorption routes. All nanoparticles were distributed in the Peyer’s patches, and the green fluorescence was stronger in the pW-LPN group than the W-LPN (Fig. 5a). One hour after oral gavage with pHPMA-nanoparticles, the absorption in the Peyer’s patches markedly increased (Fig. 5b).

### Pharmacokinetics studies

Plasma SLB and CT concentration–time profiles after oral administration of S/C-suspension, S/C-LPNs, S/C-W-LPNs, and S/C-pW-LPNs are shown in Fig. 5c. Mean pharmacokinetic parameters are listed in Additional files 1: Tables S1 and S2. For the S/C-pW-LPNs, the plasma SLB and CT concentrations were substantially higher than other treatments. The area under the curve (AUC) of the S/C-pW-LPNs for SLB was 9,796.75 ± 846.76 μg/L∙h. This value was 15.9 × , 1.5 × , and 1.13 × higher than that measured for the S/C-suspension, S/C-LPNs, and S/C-W-LPNs, respectively. The AUC of S/C-pW-LPNs for CT was 5019.36 ± 483.16 μg/L∙h. This value was 6.48 × , 1.70 × , and 1.05 × higher than that determined for the S/C-suspension, S/C-LPNs, and S/C-W-LPNs, respectively. S/C-pW-LPN MRT and t_1/2_ for SLB were 16.50 ± 2.63 h and 22.33 ± 3.77 h. These values were 4.34 × and 5.43 × higher than those calculated for the suspensions. S/C-pW-LPN MRT and *t*_1/2_ for CT were 9.23 ± 0.79 h and 10.40 ± 5.56 h. These values were 5.34 × and 3.28 × higher than those calculated for the suspensions. The relative bioavailabilities of SLB and CT in the S/W-pW-LPNs were 15.5 × and 11.4 × higher than those in the suspensions, respectively. Hence, the pW-LPNs enhanced oral bioavailability.

### Anti-metastasis studies in vivo

**Fig. 6:**
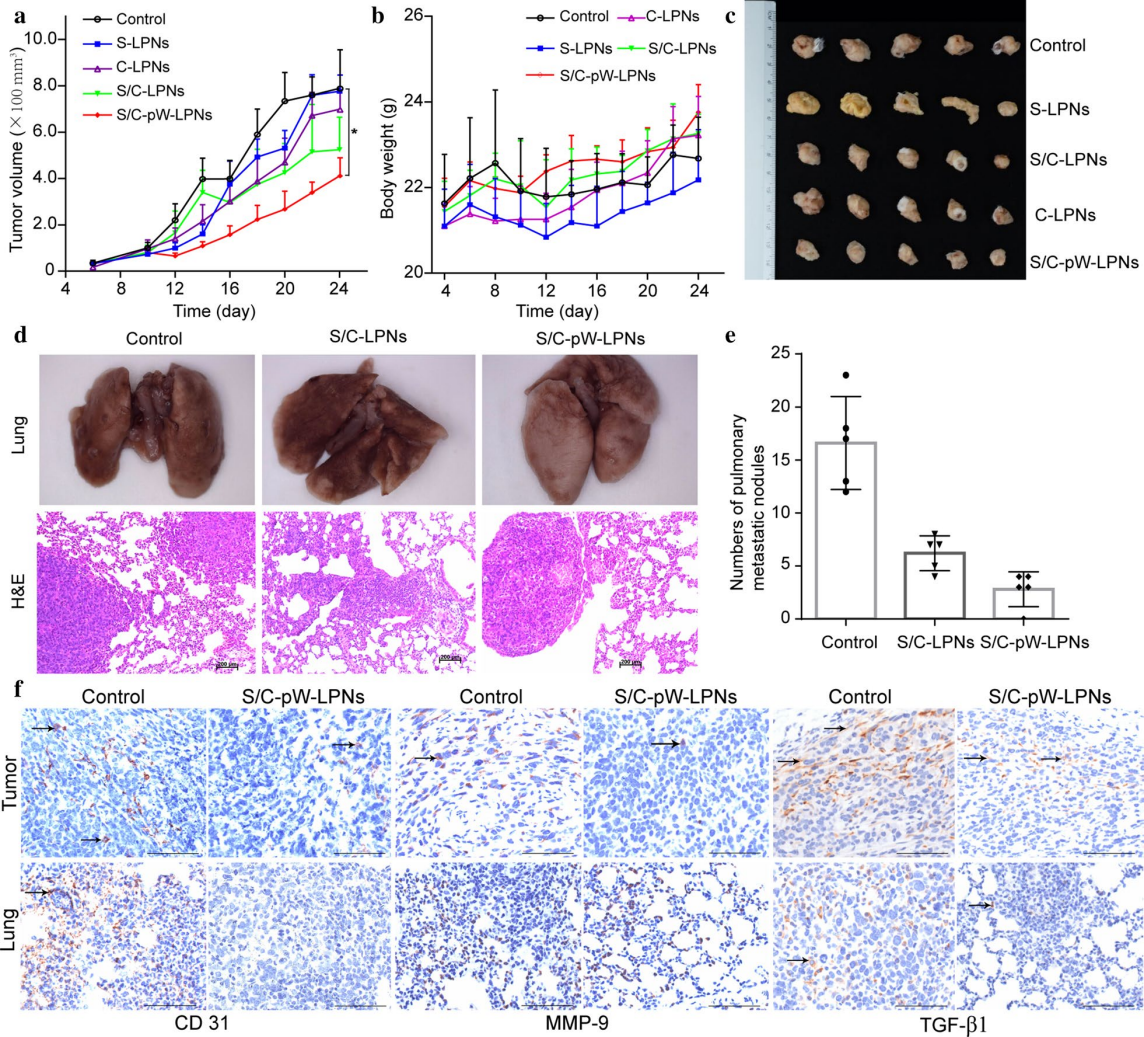
**a**–**c** In vivo antitumor effects. **a** Tumor volume in 4T1 tumor-bearing nude mice treated with control, S-LPNs, C-LPNs, S/C-LPNs, and S/C-pW-LPNs; *P < 0.05. **b** Body weight change in nude mice (mean ± SD, n = 5). **c** Tumor photographs. **d**–**f** in vivo anti-metastasis activity in 4T1 lung metastasis model. **d** Lungs of nude mice treated with control, S/C-LPNs, and S/C-pW-LPNs; H&E staining of lung tissue sections. **e** The number of surface metastases in the lung tissue (mean ± SD, n = 5). **f** Immunohistochemical (IHC) staining of CD 31, MMP-9, and TGF-β1 in tumor and lung tissue sections treated with control and S/C-pW-LPNs (scale bar = 200 μm).

The ability of S/C-W-LPNs to inhibit tumor growth was evaluated in nude mice induced with 4T1 breast cancer cells. Figure 6a shows that the S/C-LPN-treated mice exhibited smaller tumor volumes than those administered S-LPN and C-LPN. However, they did not significantly differ from each other or the saline treatment. All treatments failed to inhibit rapid tumor growth. Nevertheless, tumor growth was comparatively slower in the S/C-pW-LPNs treatment throughout the trial period. Compared with the saline control, the S/C-pW-LPNs-treated mice exhibited slower tumor growth progression. However, we observed no overall tumor regression (Fig. 6c). Although the S/C-pW-LPNs had greater antitumor efficacy than the other formulations, none of them had strong enough tumor growth suppression ability to affect tumor regression. Figure 6b demonstrates that none of the aforementioned preparations caused any critical weight loss in the mice.

As the nanoparticle formulations substantially inhibited 4T1 cell migration and invasion in vitro, their suppression efficacy was examined in a 4T1 lung metastasis model in vivo. As a result, excised lung tissue morphology was observed. Figure 6d reveals dramatic surface pulmonary metastasis in the saline control group. The results of the histopathological analysis were consistent with the morphological observation. Hematoxylin and eosin (H&E) staining of the lung tissue disclosed > 10 metastases. Unlike their limited efficacy at suppressing breast cancer tumor growth, S/C-LPNs and S/C-pW-LPNs considerably reduced the number of lung metastasis foci (Fig. 6e). However, S/C-pW-LPNs exhibited superior anti-metastasis activity. Lung tissue treated with S/C-pW-LPNs presented with smooth surfaces and minimal metastatic foci. H&E staining demonstrated that S/C-pW-LPNs more strongly suppressed the formation of metastatic foci in lung tissue than other treatments.

An immunoblot assay was conducted to elucidate the molecular mechanism of S/C-pW-LPN anti-metastasis action. Based on previous pharmacological studies of SLB and CT anti-metastasis, and the roles of specific indicators in inducing breast cancer metastasis in tumor microenvironments, CD31, MMP-9, and TGF-β1 expression were detected in breast cancer and lung metastasis tissue. As shown in Fig. 6f, the expression levels of CD31, MMP-9, and TGF-β1 in the tumor tissues of the mice treated with saline were higher than those in the tumor tissues of the mice treated with S/C-pW-LPNs. Lung tissue treated with S/C-pW-LPNs did not express CD31, and only sporadically expressed TGF-β1 and MMP-9 at the cellular level, at lower levels than the control group. Hence, the anti-metastatic action of S/C-pW-LPNs may have comprised the reduction in tumor cell adhesion to endothelial cells, inhibition of tumor angiogenesis, and attenuation of EMT.

## Discussion

In our previous study, we tested WGA-LPNs coated with hydrophilic PEG as an oral carrier and found that its mucus penetration, absorption, and antitumor efficacy were strong [23]. Functional polymer-lipid hybrid nanoparticle associated with a detachable hydrophilic polymer was developed in this study for the oral co-delivery of two active ingredients derived from traditional Chinese medicine (TCM) known to suppress breast cancer pulmonary metastasis. The pHPMA promoted WGA-LPN mucus penetration in vitro and in vivo. The hydrophilic coating on PEGylated WGA-LPNs enabled the dissociation of pHPMA from the nanoparticles and permitted WGA-mediated target cell uptake without compromising the interaction between WGA-LPNs and intestinal enterocytes. Mucin binding may alter the WGA-LPN endocytosis pathway. The nanodispersion systems developed here substantially improved SLB and CT bioavailability, enhanced their mean retention times in vivo, and augmented the delivery of a therapeutically effective drug. WGA-LPNs loaded with SLB and CT only weakly inhibited breast cancer tumor growth. However, oral administration of this complex to 4T1 tumor-bearing nude mice strongly inhibited pulmonary metastasis by downregulating CD31, MMP-9, and TGF-β1 in the tumor tissue. Therefore, the treatment mentioned above suppresses breast cancer metastasis by inhibiting factors that promote cancer progression in the tumor microenvironment.

Efficient nanocarrier penetration through the mucus layer and enterocyte uptake and transport are prerequisites for the robust anti-metastasis effect of orally administered drugs. WGA may target NAG and sialic acid on the surfaces of enterocytes and microfold cells and induce receptor-mediated endocytosis. However, WGA-LPNs encountered at least two obstacles before they arrive at the target site. First, interactions between the hydrophobic surfaces of the nanoparticles and the hydrophobic regions of the mucus hindered WGA-LPN diffusion through the mucus layer. Second, specific glycosylated components in the mucus layer trapped WGA-LPNs. Therefore, we coated the WGA-LPNs with pHPMA to protect the nanoparticles as they penetrated the mucus layer and expose the WGA functional group when the nanoparticles reach the enterocyte surfaces. The association between pHPMA and WGA-LPNs increased the hydrophilic radius of the dispersion systems, augmented surface hydrophilicity, and facilitated nanoparticle penetration through the mucus. Based on these results, an in vitro mucus penetration test and an in vivo rat jejunum distribution assay were conducted. The pW-LPNs markedly enhanced nanoparticle diffusion through the mucus because there was a low affinity between the pW-LPNs and the mucin. Moreover, pHPMA detachment from the WGA-LPNs before endocytosis was visualized. This mechanism had a dramatic impact on intestinal absorption and the subsequent biological behavior of the nanoparticles.

Mucins are the main components of mucus. They are dynamically secreted to the apical surface by goblet and Paneth cells. In our previous work, we reported that the cellular uptake of WGA-LPN-mucin complex was superior to that of WGA-LPNs alone [23]. Thus, when WGA-LPNs near the cell membrane encounter new mucin, they interact with it and the resultant complex influences nanoparticle endocytosis. Here, we sought to establish whether mucin-WGA-LPN binding depends on mucin concentration and determine its effects on the endocytosis pathways. It was found that relatively lower mucin concentrations considerably enhanced cellular nanoparticle uptake, whereas higher mucin concentrations did not. One possible explanation for this discrepancy is the large molecular size and spatial structure of mucin. The concentration of mucin influences its aggregation and physical properties [28]. At lower mucin concentrations tested here, spaces were available around the W-LPN surfaces for mucin to bind and form complexes, get recognized by enterocytes, and induce endocytosis. Mucin that is adsorbed to nanoparticle surfaces forms coronas and aggregates [29]. The resultant alterations in surface morphology may also enhance cellular uptake.

Further investigation is required to elucidate the surface changes that occur when W-LPNs and mucins interact. At higher mucin concentrations, there was little room for additional mucin, and the unbound excess mucin either self-aggregated or clustered with other components in the mucus and increased viscosity. Mucin binding on the surface might enhance cellular uptake. In contrast, excess mucin could form large aggregates, increase ambient viscosity, and obstruct nanoparticle access to the cell surface. The outcome of these two opposing processes is that there is no significant net alteration in cellular nanoparticle uptake.

The influences of mucin on the endocytosis pathway are shown in Fig. 3d. In the presence of NaN_3_, there was no substantial difference in nanoparticles with or without mucins because NaN_3_ reduced the available metabolic energy [30]. Nystatin is a lipid raft/caveola inhibitor and disruptor. Lipid rafts are microdomains consisting of dynamic protein-lipid assemblies. The lipids and proteins can be associated via hydrophobic membrane-spanning sequences, transmembrane domains, hydrophobic regions, and protein–protein and protein-lipid interactions [31, 32]. Therefore, mucin bound with nanoparticles might facilitate W-LPN endocytosis via lipid rafts, reduce the inhibitory action of nystatin, and increase cellular nanoparticle uptake compared to that observed with the mucin-free treatment. Macropinocytosis forms large vesicles that contain the nanoparticles [33]. The endocytotic mechanism of macropinocytosis is a lipid raft/caveolae-dependent, clathrin-independent pathway. Mucin bound with nanoparticles could also facilitate cellular WGA-LPN uptake by macropinocytosis. Overall, mucin bound with W-LPNs may interact with the domain associated with lipid raft/caveolae and the macropinocytosis pathway.

The natural products in medicinal herbs and/or their derivatives are promising prophylactic and therapeutic approaches to breast cancer metastasis. They are anti-metastatic via multiple targets and pathways [34–36]. SLB regulates EMT, protease activation, and other processes [37, 38]. CT inhibits tumor cell invasion, metastasis, and proliferation and promotes tumor cell apoptosis [15, 39]. Recent nanocarrier studies focused on the anti-metastatic action of SLB alone and SLB combined with chemotherapeutic and/or photothermal agents [40–42]. Breast cancer metastasis is a complex process involving multiple steps and cytokines. Thus, it was expected that SLB/CT co-administration would enhance the overall anti-metastasis effect via an interaction between two different modes of action. Relative to SLB combined with other chemotherapeutic agents, SLB/CT co-delivery was expected to enhance the inhibition of metastasis without increasing toxicity or adverse reactions. LPNs co-loaded with SLB/CT were relatively more effective than single drug-loaded LPNs at inhibiting 4T1 cell migration and invasion. For the in vivo anti-metastasis assay on orally administered nanoparticles in nude mice bearing 4T1 tumors, SLB- or CT-loaded LPNs were not effective in inhibiting tumor growth. This result was consistent with that of a previous report [40]. SLB/CT-co-loaded LPNs more potently inhibited tumor growth than nanoparticles charged with either SLB or CT alone. Nevertheless, an increase in primary tumor size is not strictly correlated with metastasis [43]. A fundamental objective of the present study was to inhibit breast cancer metastasis safely and effectively. The  LPNs co-loaded with SLB/CT substantially inhibited breast cancer metastasis. However, this effect was markedly more substantial with SLB/CT-co-loaded pW-LPNs than it was with LPNs co-loaded with SLB/CT. Tumor cells have been a primary focus of cancer research over the past four decades. However, there is emerging evidence that alterations within the tumor microenvironment, complex cell mixtures, secretory and cell surface proteins, and ECM play essential roles in tumor progression, metastasis, and therapeutic response [44, 45]. For these reasons, one target of the present study was the tumor microenvironment rather than cytotoxic therapies.

To understand how oral nanoparticles influence the tumor microenvironment, we investigated anti-metastasis in terms of angiogenesis, EMT, and ECM remodeling via immunohistochemistry (IHC) and detection of the CD31, TGF-β1, and MMP-9 expression levels. CD31 expression was quantified to determine microvessel density [46]. TGF-β1 was measured to assess EMT [47]. MMP-9 was evaluated because it regulates ECM degradation, which, in turn, facilitates breast cancer invasion and migration [48]. Upregulated MMP-9 is associated with the onset of metastases and poor prognosis. Compared with the control group, the three aforementioned biomarkers were dramatically downregulated in the tumor tissues and lungs of the pW-LPNs treatment group. Therefore, reductions in vascular density, EMT, and ECM degradation could all contribute to the anti-metastasis action of pW-LPNs. In this study, the improved anti-metastasis effect of S/C-pW-LPNs was shown in comparison with S/C- LPNs. Further studies are required to reveal the superiority of S/C-pW-LPNs in anti-metastasis in comparison with other preparations or administration routes, such as co-administration of SLB and CT without nanoparticles, single drug-loaded pW-LPNs, and parenteral administration. Breast cancer metastasis is highly complex and involves numerous processes and bipotential molecular and cellular switches [49]. It remains to be determined whether pW-LPNs mediate alternate regulatory mechanisms such as crosstalk among MMP-9, VEGF, and other molecular targets.

We hope that the orally administered nanoparticle-natural plant product formulation developed here will clinically enhance anti-metastasis in breast cancer treatment, reduce toxicity and side effects, and improve patient compliance during therapy.

## Conclusions

Here, we evaluated the effectiveness of an oral nanocarrier at delivering the two natural plant products, SLB and CT, to inhibit breast cancer lung metastasis. The nanoparticle architecture improved SLB and CT mucus penetration, cellular uptake, and oral bioavailability. Enhanced SLB and CT uptake by target 4T1 cancer cells inhibited in vitro invasion and migration. An in vivo anti-metastasis study confirmed that these novel nanoparticles co-loaded with SLB and CT inhibited lung metastasis in 4T1 breast cancer-bearing nude mice. Thus, SLB- and CT-coloaded pW-LPNs may be a practical and innovative treatment option for breast cancer lung metastasis.

## Materials and methods

### pW-LPN preparation and characterization

W-LPNs were prepared according to a previously reported method [30]. Briefly, LPNs were obtained using a modified nanoprecipitation method. SLB, CT, and PLGA were dissolved in acetonitrile. Lipoid S100 and DSPE-PEG2000 were dissolved in ethanol and dispersed in deionized water at 65 °C. The organic phase was added to the aqueous phase, and the mixture was gently stirred for 2 h. The acetonitrile was removed by rotary evaporation, and the dispersion was filtered using a 0.8-μm membrane. WGA-DOPE was prepared using a previously reported method [30]. LPNs were then incubated with WGA-DOPE for 18 h and purified using Sepharose™ CL-4B to obtain W-LPNs. pW-LPNs were formed by incubating the W-LPN dispersion in pHPMA solution (2.5 mg/mL) for 2 h. pHPMA was synthesized using a previously reported method [26]. Coumarin-6 loaded nanoparticles were prepared using the same method with coumarin-6 instead of drugs.

Particle size and zeta potential were measured using a Zetasizer Nano ZS90 (Malvern Instruments, Malvern, UK). Particle morphology was characterized by observation via TEM (JEM-2100F; JEOL Ltd., Tokyo, Japan). The conditions of the XRD analysis were as follows: Radiation source, Cu/Kα; working voltage, 40 kV; current, 100 mA; scanning range, 2θ from 3° to 50°; scanning speed, 8°/min.

### Mucus penetration assay

Mucus penetration was determined according to a previously reported method [22]. Schematic of this process is shown in Fig. 2a. Six hundred μL of mucus were placed in each 6-mm silicon tubes to a height of 30 mm. Then, 100 µL of COU-W-LPNs  and COU-pW-LPNs was gently set on the mucus surfaces, and the tubes were sealed. The tubes were kept upright, gently shaken for 12 h at 37 °C, freeze-dried at − 80 °C, and cut into 2-mm-thick slices. Each slice was transferred to a centrifuge tube and sonicated in 200 µL acetonitrile for 15 min. The suspensions were centrifuged at 1800*g* for 5 min at 4 °C, the supernatants were withdrawn, and the fluorescence intensities were measured in a microplate reader (Synergy HT; BioTek, Winooski, VT, USA) at an excitation wavelength of 485 nm and an emission wavelength of 528 nm.

In vitro penetration of pW-LPNs into HT29-MTX-E12 cell mucus was evaluated using confocal microscopy. HT29-MTX-E12 cells were seeded on a glass bottom culture dish at a density of 8 × 10^5^ per plate. After 5 days of culture, the dual fluorescent-labeled nanoparticles were incubated together with the cells for 1 h, washed thrice with phosphate-buffered saline (PBS), subjected to Hoechst33342 nuclear staining, and observed under TCS SP8 confocal system (Leica, Mannheim, Germany). Consecutive parallel xy-sections were used as focal planes along the z-axis mode at 10-μm intervals. Dual fluorescent-labeled pW-LPN composed of TRITC-labeled pHPMA and FITC-labeled W-LPNs, in which TRITC-labeled pHPMA and FITC-labeled WGA-DOPE were synthesized according to the existing procedure in the literature [50, 51].

### Nanoparticle tracking analysis

QDs (CdSe/ZnS)-loaded W-LPNs and pW-LPNs were prepared as previously mentioned [23]. Chloroform was removed from QDs dispersion (1 mg/mL) under a nitrogen stream. After redispersion in 1 mL of acetonitrile and sonification at 100 W for 5 min, they were added into the oil phase. Then, the remaining procedure was the same as W-LPNs and pW-LPNs preparation. CdSe/ZnS-loaded W-LPNs or pW-LPNs were incubated with porcine mucus (1:6, v/v) at 37 °C for 0.5 h. The mixtures were diluted with deionized water (1:10,000), and the particle concentrations and intensities were analyzed in a Nanosight NS300 (Malvern Instruments, Malvern, UK). Mucus-free samples were prepared by diluting CdSe/ZnS-loaded nanoparticles with deionized water.

### Cellular uptake

Caco-2 and HT29-MTX-E12 cells were seeded in 96-well plates at a density of 10^4^ per well and cultured in high-glucose Dulbecco’s modified Eagle’s medium (DMEM), containing 10% fetal bovine serum and 1% penicillin–streptomycin for 6 days. The cultures were equilibrated with Hanks’ balanced salt solution (HBSS) at 37 °C for 30 min, and the HBSS was replaced with DMEM containing equal volumes of the tested sample (containing coumarin-6, 1.5 μg/mL). Coumarin-6-loaded nanoparticles (LPNs and W-LPNs) were dispersed in PBS until equal fluorescence intensities were achieved. Then mucin solution (0.025% w/v) or PBS was added. After 1 h incubation, the cells were washed thrice with PBS and lysed using CelLyti M (Sigma-Aldrich, St Louis, MO, USA). Fluorescence intensities were detected using a microplate reader at 485 nm excitation and 525 nm emission. The mucus layer was removed from the cells by preincubation with 10 mM *N*-acetyl-*l*-cysteine. The nanoparticle dispersions were combined with mucin solution, and the mixtures were gently stirred at 37 °C for 3 h to elucidate the influence of mucin on cellular uptake. After ultrafiltration using Vivaspin 6.0 (MW1000KD, Sartorius Stedim Biotech SA, Aubagne, France) at 3000*g* for 20 min at 20–25 °C, the filtrate was collected, and cellular uptake was observed under a confocal laser scanning microscope. Nile Red and FITC were used to label the nanoparticles and mucin, respectively. The nanoparticles were incubated with FITC-mucin (0.025% w/v) with gentle stirring at 37 °C for 1 h. The sample was purified using a 0.8-μm nylon membrane filter and ultrafiltered using a Vivaspin 6.0 at 3000*g* for 20 min at 20–25 °C. The sample was redispersed in PBS (pH 7.4). Caco-2 cells were seeded at a density of 5 × 10^4^ well^−1^ and cultured overnight. Before the experiment, the culture medium was withdrawn, and the cells were incubated for 1 h in DMEM containing nanoparticles with or without mucin, washed thrice with PBS, fixed with 4% (*v*/*v*) paraformaldehyde (PFA), subjected to 4′,6-diamidino-2-phenylindole (DAPI) staining for 15 min, and washed thrice with PBS. Cells were observed under a Leica TCS SP8 confocal system (Leica Microsystems, Wetzlar, Germany).

### In vitro cell migration and invasion assay

To evaluate cell migration, we plated 10^5^ 4T1 cells in serum-free medium were plated in the top chamber of a Transwell. C-W-LPNs, S-W-LPNs, and S/C-W-LPNs (5.0 μg/mL CT; 30 μg/mL SLB) were dispersed into the top chamber. To evaluate cell invasion, we plated 10^5^ 4T1 cells in serum-free medium in the top chamber of a Transwell pre-coated with Matrigel (BD Biosciences, Franklin Lakes, NJ, USA). Nanoparticles identical to those used in the cell migration study were dispersed into the top chamber. Complete medium was added as a chemoattractant to the lower chamber. After incubation for 24 h at 37 °C, cells failing to migrate to or invade the upper chamber were carefully removed with a cotton swab. Cells that traversed the membrane were fixed with 90% (v/v) ethanol, stained with Hematoxylin-eosin, and observed under the microscope (Leica DMi1, Leica, Mannheim, Germany).

### In vivo bioavailability

Sprague–Dawley rats (female) each weighing 220 ± 20 g were randomly assigned to four groups (n = 5). S/C-suspension, S/C-LPNs, S/C-W-LPNs, and S/C-pW-LPNs were administered by oral gavage, containing equivalent doses of SLB and CT (80 mg/kg). The S/C-suspension was prepared by dissolving equimolar SLB and CT in CMC-Na dispersion. Four hundred μL of blood samples were withdrawn at 30 min, 45 min, 60 min, 90 min, 2 h, 3 h, 5 h, 8 h, 12 h, and 24 h and placed in heparinized tubes. For one period of 24 h, two animals were used to withdraw the blood samples, one for the first five time points, and the other is for the remaining five time points. After centrifugation at 3500*g* and 4 °C for 10 min, the plasma was separated. SLB in plasma was determined according to a previously reported method [52]. The plasma samples were treated with β-glucuronidase to liberate SLB. The free SLB was extracted using tert-butyl methyl ether, and the organic layer was evaporated with a nitrogen stream. Details of SLB and CT determination are described in the Supporting Information. The pharmacokinetic parameters were obtained using a non-compartmental model in Drugs and Statistics v. 2.0 (Mathematical Pharmacology Professional Committee of China).

### In vivo antitumor efficiency

Female BALB/c nude mice aged 5–6 weeks were randomly assigned to four groups (n = 5). Each mouse was injected with 4 × 10^5^ 4T1 cells in the second pair of mammary pads. After 3 days, the mice were orally administered saline, S-LPNs, C-LPNs, S/C-LPNs, or S/C-pW-LPNs containing equivalent doses of SLB and CT (80 mg/kg). The treatments were applied every 2 days. Tumor volume obtained using calipers and body weight were measured every second day. Tweenty three days after the first administration, all mice were sacrificed, the tumors and lung tissues were excised and photographed, and the pulmonary metastases were counted. H&E staining was performed on the lung tissues for histological examination (n = 3). Immunohistochemical (IHC) analysis was conducted to measure the CD31, TGF-β1, and MMP-9 expression levels in the tumor and lung tissues. Tumor and lung tissues were fixed in formalin, embedded in paraffin, cut into 5 μm thick sections using a microtome (Leica CM1510, Mannheim, Germany), baked at 60 °C, deparaffinized in xylene, and rehydrated in a graded ethanol series and distilled water. Antigens were retrieved using high temperature–pressure repair with Tris-ETA buffer. After four rinses with PBS, the sections were blocked with hydrogen peroxide (H_2_O_2_), washed 4× with PBS, incubated with goat serum for 40 min, and incubated with rabbit monoclonal anti-MMP-9 antibody (ab22802; Abcam, Cambridge, UK), rabbit monoclonal anti-CD31 antibody (ab182981; Abcam, Cambridge, UK), and rabbit monoclonal TGF–β1 antibody (ab215715; Abcam, Cambridge, UK) in a dark chamber at 4 °C overnight. The sections were then washed in PBS, incubated with horseradish peroxidase (HRP)-goat anti-rabbit secondary antibody (MaxVision™ HRP-polymer anti-rabbit IHC kit, Fuzhou Maixin Biotech. Co. Ltd., Fuzhou, China) of the appropriate species at 37 °C for 30 min and washed with PBS. Staining was performed with the chromogenic substrate 3,3′-diaminobenzidine (DAB). The sections were then rinsed under running water for 10 min, counterstained with hematoxylin, and washed under running water. After dehydration with ethanol, the sections were mounted on glass slides and visualized under a microscope.

### Statistical analysis

Data are represented as mean ± standard deviation (SD). One-way analysis of variance with Tukey’s post-test was used for data comparisons. P < 0.05 was considered statistically significant.

## Supplementary information

**Additional file 1:** Functional oral nanoparticles for delivering silibinin and cryptotanshinone against breast cancer lung metastasis.

## Data Availability

All data generated and analyzed in this study are included in this manuscript.

## Abbreviations

AUC: Area under the curve
CCK-8: Cell counting kit 8
CD31: Cluster of differentiation 31
CLSM: Confocal laser scanning microscopy
CT: Cryptotanshinone
DAB: 3,3′-diaminobenzidine
DAPI: 4′,6-diamidino-2-phenylindole
DMEM: Dulbecco’s modified Eagle’s medium
ECM: Extracellular matrix
EMT: Epithelial-to-mesenchymal transition
FITC: Fluorescein isothiocyanate
H&E: Hematoxylin and eosin
HBSS: Hanks’ balanced salt solution
IC_50_: Half-maximal inhibitory concentration
HRP: Horseradish peroxidase
IHC: Immunohistochemistry
LPN: Lipid-polymer nanoparticles
MMP-9: Matrix metallopeptidase 9
MRT: Mean residence time
NAG: *N*-acetyl-*d*-glucosamine
PBS: Phosphate-buffered saline
PEG: Polyethylene glycol
PFA: Paraformaldehyde
pHPMA: Poly-*N*-(2-hydroxypropyl) methacrylamide
QD: Quantum dot
SLB: Silibinin
t_1/2_: Half-life
TCM: Traditional Chinese medicine
TEM: Transmission electron microscopy
TGF-β1: Transforming growth factor beta 1
TRITC: Tetramethylrhodamine
VEGF: Vascular endothelial growth factor
WGA: Wheat germ agglutinin
XRD: X-ray powder diffraction
