# Supplementary material for: Functional oral nanoparticles for delivering silibinin and cryptotanshinone against breast cancer lung metastasis
Source: J Nanobiotechnology. 2020 May 30;18:83. doi: 10.1186/s12951-020-00638-x (PMC7260741; doi:10.1186/s12951-020-00638-x)
Supplement: Supplementary file 1 — Additional file 1: Functional oral nanoparticles for delivering silibinin and cryptotanshinone against breast cancer lung metastasis. [file 12951_2020_638_MOESM1_ESM.docx]

**Additional file**

**Functional oral nanoparticles for delivering silibinin and cryptotanshinone against breast cancer lung metastasis**

**Ying Liu^1^, Xingmei Xie^1^, Xuefeng Hou, Junyi Shen, Jiangpei Shi, Haizhen Chen, Yuanzhi He, Zhi Wang, Nianping Feng***

School of Pharmacy, Shanghai University of Traditional Chinese Medicine，Shanghai 201203, China

***Materials***

Poly(lactic-co-glycolic acid) (PLGA， 50:50, Mw 7,000–17,000 or Av. MW), silybin (SLB, purity > 98%) and cryptotanshinone （CT, purity > 98%）were provided by Dalian Meilun Biotechnology Co. Ltd (Dalian, China). Lipoid S100 was purchased from AVT Technology Co. (Shanghai, China). HPMA monomer, fluorescein isothiocyanate (FITC), tetraethyl rhodamine isothiocyanate (TRITC), nile red, DAPI, coumarin-6 and Type II mucin from porcine stomach were obtained from Sigma-Aldrich Co. (MO, USA). Hydrophobic CdSe/ZnS quantum dots (QDs) (2-5 nm) were purchased from Mukenano. Co. Ltd. (Nanjing, China). Both Caco-2 cells and 4T1 cells were obtained from Shanghai Institutes for Biological Sciences (Shanghai, China). HT29-MTX-E12 were obtained from the European Collection of Authenticated Cell Cultures (ECACC, Salisbury, UK, Catalog Number: 12040401).

**Methods**

*Encapsulation efficiency and drug loading determination*

The nanoparticle dispersion was mixed with acetonitrile (1:4, *v*/*v*) using sonication for 10 min.

After filtration with 0.22 μm filter membrane, 20 μL filtrate was analyzed using HPLC to determine SLB and CT concentration. Encapsulation efficiency (EE) and drug loading (DL) were calculated according to equation 1 and 2, respectively.

| $EE(\%)=\frac{W_{N}}{W_{D}}\times100$ | | (1) | |
| --- | --- | --- | --- |
| $DL(\%)=\frac{W_{N}}{W_{T}}\times100\%$ | | (2) | |

Where W*_N_* is the amount of drug incorporated into nanoparticles, W*_D_* is the initial total amount of DL, W*_T_* is the total weight of nanoparticles.

HPLC was performed on an Agilent 1260 system (Agilent Technologies, Santa Clara, CA, USA). HPLC analysis for silibinin was performed using Platisil ODS column (C18; 250 × 4.6 mm, 5 μm; Dikma Technology, Shanghai, China). The mobile phase was (A) acetonitrile, (B) methanol and (C) water (containing 1% formic acid) (A:B:C = 9.6:40.4:50, *v*/*v*/*v*). Other conditions include a flow rate of 0.8 mL/min and a detection wavelength of 288 nm. The HPLC condition for CT was as follows: Mobile phase of water and acetonitrile (20:80, *v/v*); flow rate of 1 mL/min; detection wavelength of 270 nm.

*In vitro release study*

In vitro SLB and CT release from pW-LPNs were evaluated using a dialysis method. Briefly, 1 mL of nanoparticle dispersion was added to a pretreated dialysis bag (molecular weight cut-off of 14 kDa). The release study was performed at pH 6.8 simulated intestinal fluid (without enzyme) containing 1% Tween 80 at 37 ± 1 ℃ shaking at 100 strokes per min. 1 mL of liquid in the release medium was withdrawn at 0.5, 1, 2, 4, 6, 8, 12, 24, 30, and 48 h, and an equal volume of fresh release medium was supplemented. SLB and CT levels were determined using HPLC.

*Cell viability assay*

Cell viability assay was conducted following a previously published method ^1^. 4T1 cells were seeded into 96-well culture plates at a density of 8000 cells/100 μL/well. The cells were incubated for 24 h at 37 °C and 5 % CO_2_. After the removal of the culture medium, the cells were treated with various concentrations of S-W-LPNs, C-W-LPNs, S/C-W-LPNs in triplicate. After 24 h incubation, the medium was replaced with culture medium containing 10% (*v*/*v*) Cell Counting Kit-8 (CCK-8) (Sigma-Aldrich, USA). The cells were cultured for an additional 1 h. Then, the absorbance of each well was read at 450 nm using a microplate reader (Synergy HT; BioTek, Winooski, VT, USA). Cell viability was calculated using the following equation:

$$Cell viability rate\left( \% \right)=\frac{A_{sample}-A_{negative control}}{A_{positive control}-A_{negative control}}\times100\%$$

In which, A_positive control_ indicates the absorbance of the untreated cells, A_negative control_ is the absorbance of the blank well filled with culture medium containing 10% CCK-8 solution.

The IC_50_ values for S-W-LPNs, C-W-LPNs, S/C-W-LPNs were obtained using GraphPad

Prism 7.0 (GraphPad Software, USA).

*In vitro wound healing migration assay*

The cells were seeded in 6-well plates at a concentration of 5 × 10^5^ cells/well and grown to confluence. A scrape through the confluent monolayer was made using a sterile 200 μL pipette tip. After washing twice with PBS, the cells were treated with the serum-free DMEM containing C-W-LPNs, S-W-LPNs, S/C-W-LPNs (CRY 5.0 μg/mL, SLB 30 μg/mL) or without nanoparticle dispersions and cultured for 24 h. The cell migration distance was observed and captured using an Olympus IX83 microscope (Olympus, Japan) at 0 h and 24 h.

*Nanoparticle distribution in the jejunum and Peyer’s patches of rats*

Nanoparticle distribution in the jejunum of rats was investigated following a previously reported method^2^. Six healthy Sprague-Dawley (SD) rats (weighing 220 ± 20 g, half male and half female) were used. Before the experiments, the animals were fed a liquid diet for 24 h followed by fasting for 24 h. Coumarin-6-loaded W-LPNs and pW-LPNs dispersions (100 μL each) were fed to the rats by oral gavage. The rats were sacrificed 1 h after the gavage, and their jejunum was excised. After gentle removal of the excess nanoparticles, approximately 0.5 cm of jejunum was embedded and mount fixed in optimal cutting temperature compound, and flash-frozen in liquid nitrogen. Cryostat tissue sections of the jejunum (10-μm-thick) were cut using CM3050S cryostat (Leica Microsystems, Welzlar, Germany). Further, after 4',6-diamidino-2-phenylindole (DAPI) staining, the sections were observed under the DP80 microscope (Olympus, Tokyo, Japan) using a wavelength of 485 nm for excitation and 528 nm emission.

Nanoparticle distribution and absorption in Peyer’s patches were evaluated as follows^3^: 6 healthy SD rats (weighing 220 ± 20 g, half male and half female) were fasted overnight before the experiment, with free access to water. The animals were anesthetized intraperitoneally with chloral hydrate. The intestinal Peyer’s patches were taken, followed by washing with saline, injection of coumarin-6-loaded W-LPNs and pW-LPNs dispersions and ligation. The rats were sacrificed after 1 h, and the ligated section was excised. After washing the lumen surface and gentle removal of the nanoparticle dispersions, the Peyer’s patches were collected. Similar with the methods used to determine the nanoparticle distribution in the jejunum, the loop was fixed in 4% paraformaldehyde, followed by embedding, freezing in liquid nitrogen, producing 6-μm thick cryostat tissue slices and observation under the microscope. The quantitative analysis was conducted as follows: The collected Payer was blotted dry. Then, they were homogenized and incubated in RIPA lysis buffer (Thermo Scientific, Rockford, USA). The lysate was further centrifuged at 11700 × *g* for 3 min. A microplate reader (BioTek Instruments, Winooski, USA) was used to measure the fluorescence intensity in the supernatant at 485 nm. The protein content in the supernatant was determined using a bicinchoninic acid (BCA) assay.

*Plasma concentration of SLB and CT measurement by HPLC*

HPLC chromatographic condition was the same as in vitro HPLC determination as mentioned above. The sample preparation for plasma content determination was as follows: An aliquot of 100 µL plasma samples was placed into 1.5-mL microcentrifuge tubes, followed by the addition of 333 μL pH 5.0 PBS and 125 U 77.5 μL β-glucuronidase. Then, it was vortexed for 1 min and incubated at 37 ℃, shaking, in a water bath for 18 h. 66.5 μL of 1.0 M sodium carbonate solution and 332.5 μL of pH 8.0 borate buffer solution were added and vortexed for 30 s. Four mL of tert-Butyl methyl ether was added, followed by vertexing 5 min. After centrifugation at 1800 × *g* for 10 min, the supernatant was withdrawn and dried under a nitrogen stream. The residues were redispersed using the mobile phase, vortexed for 1 min and centrifuged at 3000 × *g* for 10 min. The SLB content was analyzed using HPLC.

An aliquot of 100 µL plasma samples was placed into 1.5-mL microcentrifuge tubes. Ethyl acetate (500 µl) was added to the tube, vortexed for 3 min and centrifuged at 3000 ×*g* for 3 min. The organic supernatant was transferred into a tube. The extraction process mentioned above was repeated using 200 µl ethyl acetate. The organic supernatant was combined and dried under a nitrogen stream. The obtained residual was resuspended in 100 µL, followed by vortexing for 1 min, and centrifugation at 3000 × *g* for 10 min. The supernatant was withdrawn for CRY content determination. The standard curve for SLB and CT were as follows: A_SLB_ = 0.0246C + 1.9059，r = 0.9990, concentration ranges 78.1~2500.0 ng/mL; A_CT_ = 0.0399C + 0.1769，r = 0.9995，concentration ranges 25.0~2500.0 ng/mL. The relative standard deviation (%RSD) for the intra-day and inter-day precisions were < 15%.

**Results**

*Encapsulation efficiency and drug loading*

The encapsulation rate for SLB and CT was 97.12±0.12% and 99.89±0.1%, respectively. The DL for SLB and CT was 10.5±0.54% and 6.12±0.1%, respectively.

*In vitro release study*

The release curve is shown in Figure S1. Both SLB and CT showed gradually increasing release profiles. The accumulative release was approximately 81% and 58% for CT and SLB, respectively. The slightly decreased accumulation of CT at 30 and 48 h was possibly due to the degradation of CT at 37 ℃. To get further and more detailed understanding of the release features, it would be beneficial to investigate the release behavior of CT and SLB from pW-LPNs in a simulated gastric fluid in future studies.

***References***

1. Yu X, Sun L, Tan L, Wang M, Ren X, Pi J, Jiang M, Li N. Preparation and Characterization of PLGA-PEG-PLGA Nanoparticles Containing Salidroside and Tamoxifen for Breast Cancer Therapy. AAPS PharmSciTech. 2020; 21:85.

2. Maisel K, Ensign L, Reddy M, Cone R, Hanes J. Effect of surface chemistry on nanoparticle interaction with gastrointestinal mucus and distribution in the gastrointestinal tract following oral and rectal administration in the mouse. J Control Release. 2015;197:48-57.

3. Liu Y, Jiang ZF, Hou XF, Xie XX, Shi JP, Shen JY, He YZ, Wang Z, Feng NP. Functional lipid polymeric nanoparticles for oral drug delivery: Rapid mucus penetration and improved cell entry and cellular transport. Nanomedicine. 2019:102075.

Additional file **Figure Legends**

Additional file Figure S1. In vitro release profiles of SLB and CT from pW-LPNs.

Table S1. Pharmacokinetic parameters of SLB in S/C-suspension, S/C -LPNs, S/C -W-LPNs and S/C -pW-LPNs.

Table S2. Pharmacokinetic parameters of CT in S/C-suspension, S/C -LPNs, S/C -W-LPNs and S/C -pW-LPNs.

Additional file Table S1

| Parameters | Suspension | | LPNs | W-LPNs | pW-LPNs |
| --- | --- | --- | --- | --- | --- |
| *t*_1/2_ (h) | 4.11 ± 2.66 | 10.57 ± 3.98* | | 20.26 ± 7.14*** | 22.33 ± 3.77*** |
| *t*_max_ (h) | 1.40 ± 0.42 | 1.90 ± 0.22 | | 1.90 ± 0.22 | 1.80 ± 0.27 |
| *C*_max_ (ng/mL) | 424.72 ±78.79 | 1787.45 ±186.67*** | | 2432.28 ± 715.347*** | 3379.44 ± 1023.53*** |
| *AUC*_0-t_ (ng• h/mL) | 525.10± 120.52 | | 5176.21 ± 1118.09*** | 6446.8 ± 1530.19*** | 8164.42 ± 1449.25*** |
| *AUC*_0-∞_(ng• h/mL) | 616.72 ± 120.47 | | 6520.53 ±1997.61*** | 8594.32 ± 1905.49*** | 9796.75 ± 846.76*** |
| *MRT_0-t_* (h) | 3.80 ± 1.32 | 11.01 ± 5.50** | | 15.06 ± 3.61*** | 16.50 ± 2.63*** |
| *MRT* _0-∞_ (h) | 6.75± 2.10 | 14.13 ± 3.34 | | 18.39 ± 5.89* | 19.07 ± 2.80* |

^†^Statistical difference compared with Suspension:^*^ *P* < 0.05, ^**^ *P* < 0.01, ^***^ *P* < 0.001

Additional file Table S2

| Parameters | Suspension | LPNs | W-LPNs | pW-LPNs |
| --- | --- | --- | --- | --- |
| *t*_1/2_ (h) | 3.17 ± 1.83 | 8.82 ± 2.83 | 9.475 ± 2.23* | 10.40 ± 5,56* |
| *t*_max_ (h) | 1.10 ± 0.38 | 2.75 ± 1.56 | 2.7 ± 0.67 | 2.00 ± 0.00 |
| *C*_max_ (ng/mL) | 164.99 ± 22.87 | 197.07 ± 25.84 | 405.59 ± 214.59* | 468.40 ± 49.98** |
| *AUC*_0-t_ (ng• h/mL) | 367.92 ± 188.9 | 2363.29 ± 88.97*** | 3915.00 ± 323.84*** | 4213.70 ± 490.46*** |
| AUC_0-∞_ (ng• h/mL) | 775.05 ± 315.77 | 2957.48 ±302.3*** | 4778.9 ± 691.6*** | 5019.36 ± 483.16*** |
| *MRT*_0-t_ (h) | 1.73 ± 0.87 | 9.06 ± 0.73*** | 9.20 ± 0.83*** | 9.23 ± 0.79*** |
| *MRT* _0-∞_ (h) | 5.06 ± 2.61 | 15.93 ± 3.06** | 15.75 ± 3.02** | 17.91 ± 4.44** |

^†^Statistical difference compared with Suspension:^*^ *P* < 0.05, ^**^ *P* < 0.01, ^***^ *P* < 0.001

Additional file Figure S1.
